# Quantitative Easing and bank risk-taking behavior

**DOI:** 10.1016/j.heliyon.2023.e17965

**Published:** 2023-07-11

**Authors:** Saroj Dhital, Chandler Dixon, Eddie Evanczyk

**Affiliations:** Economics and Business Department, Southwestern University, United States of America

**Keywords:** Quantitative Easing, Bank lending, Monetary policy, Central bank, Risk-taking channel, Reserve balance, Portfolio substitution effect

## Abstract

In this paper, we examine the effect of reserve creation due to the Federal Reserve's Large-scale Asset Purchase programs on bank lending and risk-taking behavior. In particular, we test the existence of a risk-taking channel that induces banks with higher reserve accumulation due to the Fed's policy to increase the share of riskier loans. Exploiting the heterogeneity in the exposure to such asset purchase programs, as measured by relative mortgage-backed securities holdings in the banks' portfolios, we use difference-in-differences analysis to study the effect of the Fed's policies on the supply of total loans and the share of riskier loans such as real estate, commercial and industrial, and consumer loans. We find that the first and third round of Quantitative Easing (QE) policies led to an increase in both the total loans and the share of riskier loans within the banks' portfolios, supporting the risk-raking channel. The second round of QE on the other hand, had no significant impact on banks' lending behavior.

**Table 1:** Acronyms Table.

Terminology	Acronym
Quantitative Easing	QE
Large-Scale Asset Purchase	LSAP
Commercial and Industrial	CI
Mortgage-Backed Securities	MBS
Return on Assets	ROA
Federal Open Market Committee	FOMC

Notes: The table presents the acronyms of terms used in the paper.

## 1 Introduction

A series of aggressive large-scale asset purchases (LSAPs), also known as Quantitative Easing (QE), by the Federal Reserves following the Global Financial Crisis has led economists to study the effect of such unconventional monetary policies and understand how such policies transmit throughout the economy. The effectiveness of such policies has been a topic of debate over the past decade as central banks in developed countries continue to implement the QE policies. Hence, a more complete understanding of how such policies are transmitted to the economy is necessary. There is rich theoretical literature studying the effects of reserve accumulation due to the Fed's policies on the banking sector. Empirical studies analyzing the effect of reserve accumulation due to the QE policies on banks' portfolio decisions are rare, however. Studies analyzing the effect on banks' risk-taking behavior are even rare.

In this paper, we study the effect of the increase in the reserves due to the QE policy on the banks' risk-taking behavior. To do so, we study the effect on the share of risky loans issued by the commercial banks. Studying the effect on the share of risky loans helps us understand the banks' risk taking tendencies due to the increase in reserves. Changes in these loan categories as a share of total loans helps assess the riskiness of banks' marginal lending. We study the effect on real estate loans, commercial and industrial (C&I) loans, and consumer loans. These categories of loans have relatively high delinquency rate. In addition, these loans carry regulatory risk weights of at least 100%.^1^

The study is significant for a number of reasons. First, Banks are directly affected by the QE policies as an increase in the bank reserves is the defining characteristic of such policies. Banks play an important role in facilitating economic activity and a shock to the banking sector can have a major impact throughout the economy. Hence, it is essential to understand the impacts of such policies on the banking sector. It offers valuable insights into how the Federal Reserve's monetary policies affect bank lending and risk-taking behavior. These are crucial factors that impact the stability and growth of the economy. Second, the paper explores the existence of a risk-taking channel, which has important implications for financial stability. If banks respond to the Federal Reserve's policies by taking on more risky loans, it could increase the likelihood of defaults and lead to greater instability in the financial sector. Third, the paper's findings can be used by policymakers to inform their decisions about the effectiveness and potential risks of their monetary policies. If these policies have unintended consequences, such as encouraging risk-taking among banks, policymakers may need to adjust their approach to ensure financial stability.

An increase in reserves accumulation forced by the Fed likely induces banks to seek investment in other assets such as securities and loans at a higher rate. Indeed, Friedman and Schwartz [17], for instance, argue that the new reserves created by Fed policies are larger than the optimal amount that banks previously held. As reserves increases, banks extend lending and invest in other assets in order to restore the marginal benefit of assets in the banks' portfolios. Such portfolio substitution mechanism relies on imperfect substitutability of reserves and other assets. If such an increase in reserves induces an increase in bank lending, it is probable that the marginal lending carries a higher risk than the riskiness of the existing portfolio. Indeed, for a given interest rate, banks first choose lending opportunities with higher risk-adjusted return. As banks expand their lending, the remaining lending pool becomes riskier.^2^ Hence, the portfolio substitution effect induces larger risk taking from the banks. We explore such risk-taking channel in this paper.

Studying the effects of QE poses a significant challenge, overcoming the endogeneity of banks' reserve holdings to other portfolio decisions. While the supply of total reserves is fixed by the Fed, the distribution of the reserves is determined by the individual banks. Hence, relating bank-level outcome to their reserves balances poses an endogeneity problem. To overcome this, we exploit the cross-sectional variation in the mortgage-backed securities (MBS) holding in the banks' portfolio and employ a difference-in-differences identification strategy as in Rodnyansky and Darmouni [35]. Each bank's exposure to the LSAPs is captured by the MBS as a share of total securities in the bank's portfolio (MBS-to-securities ratio) prior to the implementation of the policies. Indeed, the more MBS a bank holds, the more sensitive to the QE policy it is. As Rodnyansky and Darmouni [35] documents, there is significant heterogeneity in the MBS-to-securities ratio among the commercial banks and hence, their exposure to the LSAPs.

We find significant effects of QE1 and QE3 on banks' lending behavior. There is evidence of increased lending due to the increase in reserves among the banks with large MBS holdings relative to their counterparts as in Rodnyansky and Darmouni [35]. In addition, the share of riskier loans issued increases as a result of QE1 and QE3 as in Kandrac and Schlusche [24], indicating a shift towards riskier loans. The findings are robust. When the Fed targeted MBS in QE1 and QE3, the reserves of the commercial banks increased dramatically as banks held significant amount of the MBS in their portfolios. Such increase in reserves induced banks to shift towards riskier loans, which may suggest ‘search for yield’ among banks who experience a reduction in net interest margin due to the influx of reserves as a result of the QE policy. The findings suggest that the Fed's policy can stimulate lending and risk-taking by increasing the reserves in the banking system.

The results suggest that QE2 did not have a significant impact on bank lending as QE2 primarily focused on Treasury securities, rather than MBS, which are sparsely held by commercial banks. Such findings indicate that the effect of the Fed's policies work through a narrow channel that affect the prices of the targeted asset solely and not through a broad channel that affect all long-term rates when targeted at a particular asset such as MBS.

There are various channels through which LSAPs affect banks lending behavior. First is the *bank lending channel* as in Kashyap and Stein [25], [26], where QE effectively replaces MBS in the banks' portfolio with reserves, thereby increasing their ability to issue reservable deposits and ability to lend. Note that a necessary condition for this channel is the failure of the Modigliani-Miller proposition. Second is the *net-worth channel* as in Bernanke and Gertler [5], Kiyotaki and Moore [28], Bernanke et al. [9] and Adrian and Shin [2], where QE raises the price of MBS in the banks' books which raises the mark-to-market value of equity, or net worth. Such increase in net worth induces banks to increase lending, assuming that they target a constant leverage ratio. Third is the *liquidity channel* where QE infuses liquid asset (reserves) in exchange for MBS by the Fed, or via secondary market, without significantly altering the balance sheet of the banks. Such infusion of liquidity allows banks to expand lending. Fourth is the *signaling channel* as in Bauer and Rudebusch [4] and Krishnamurthy and Vissing-Jorgensen [29] where the Fed commits to keeping the interest rates low (and hence yield on widely traded assets) for the foreseeable future and encourage banks to extend lending. Note that the transmission channels of QE explored in this paper can operate alongside other channels explored in the literature.

Most studies thus far focus on understanding the macroeconomic effect of QE policies, such as the effect on long-term interest rates and inflation expectation, or yields on widely traded financial assets as in Gagnon et al. [18], D'Amico and King [16] and Gilchrist and Zakrajšek [19], or mechanism that work through signaling channel as in Bauer and Rudebusch [4] and Krishnamurthy and Vissing-Jorgensen [29]. There's a rich theoretical literature on the effect of reserve creation on other assets that work through portfolio substitution effect.^3^ The securities on the banks' balance sheet is swapped for reserves as a result of QE. Also, reserves increase via increase in bank deposits from non-bank institutions that previously held the securities. As Friedman and Schwartz [17] explains, creation of the new reserves induces banks to seek investment in other assets such as securities and loans at a higher rate. This is the case as the banks now have reserves higher than the optimal amount and the marginal benefit of holding the reserves are now lower. Consequently, banks will look to invest in alternative assets. Such portfolio substitution mechanism relies on imperfect substitutability of reserves and other assets.

Despite the prevalence of theoretical studies on the reserve creation due to monetary policy, empirical studies linking the reserve accumulation as a result of QE to banks' investment decision remain scarce.^4^ We contribute to this nascent literature. The main contribution of the paper is to identify the effect of reserve accumulation due to the QE policy on the share of risky loans. Studies analyzing the effect of the QE policy on Banks' risk-taking behavior are rare. Most studies in the literature focus on risk taking due to low or negative policy rates.^5^ We focus on risk taking due to reserve expansion induced by QE, which represents a gap in the literature. In addition, we contribute to the following literature. First, we contribute to the literature on the impact of monetary policy on banks' investment decision in relation to the risk-taking channel.^6^ Second, we contribute to the literature that studies the effect of QE policy. Third, we contribute to the literature on the effect of liquidity shock on credit supply.^7^ Fourth, we contribute to the literature of economic uncertainty and banking stability.^8^

Understanding the effect of reserve accumulation due to the QE policy on banks' lending behavior is essential as banks play an important role in facilitating economic activity. This paper analyzes the effect of the QE policy on banks' risk-taking behavior by studying the effect of the policy on the share of risky loans issued by the banks. We find that QE1 and QE3 raised the share of risky loans issued by the banks, indicating an increased risk taking by the banks due to the policies. We do not find any significant impact of QE2 on banks' lending behavior. The findings contribute significantly to the literature as studies analyzing the effect of the QE policy on Banks' risk-taking behavior are rare.

The remainder of the paper proceeds as follows. Section 2 outlines the data used in the analysis, section 3 describes the empirical methodology used and section 4 presents the findings of our analysis. We conclude the paper with summary of findings in section 5.

## 2 Data

The dataset used for the analysis is the Consolidated Reports of Condition and Income, FFIEC 031 and FFIEC 041 regulatory filings, commonly known as Call Reports. All commercial banks with insured deposits must file Call Reports every quarter. Call Reports include detailed information on the composition of banks' balance sheets, income statements and off-balance sheet items. We construct a set of dependent, independent and control variables used in the analysis from Call Reports for the sample period of 2008Q1 to 2014Q1. The summary statistics of variables used in the analysis is presented in the appendix.

In contrast to the approach commonly used in the empirical literature using Call Reports, we do not aggregate the data at the holding-company level. Many banks are part of multibank holding companies and non-bank subsidiaries are not included in the Call Reports. The reasons for using bank level data instead of holding-company data are twofold. First, as demonstrated by Rodnyansky and Darmouni [35], the results are largely unaffected when disaggregated data (bank level data) are used instead of aggregated data (holding-company level data). Second, there's a significant reduction in sample size when working with aggregated data.^9^

**Figure 1:**
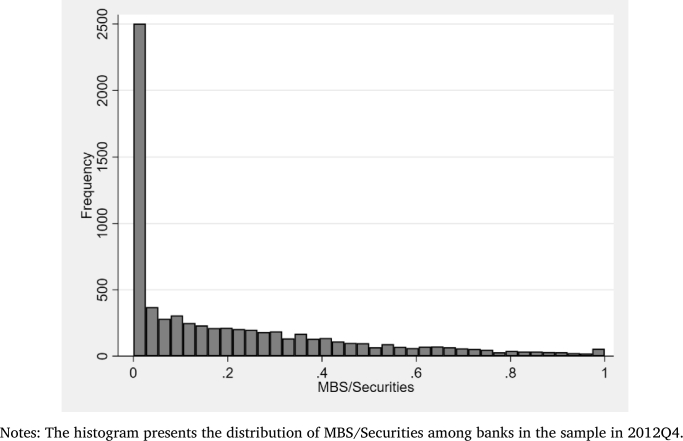
Cross-Sectional Variations in MBS/Securities.

Fig. 1 presents the histogram of the MBS-to-Securities ratio before QE3.^10^ The cross-sectional variation in MBS-to-Securities ratio is quite large over the entire sample period as illustrated by the histogram. The average MBS-to-Securities ratio during the period is 0.19 with a standard deviation of 0.38. There are several banks that have no MBS holdings. Rodnyansky and Darmouni [35] show that the relative MBS holdings are remarkably sticky throughout the sample period.^11^ Hence, the MBS-to-Securities distribution illustrated in the histogram is representative for the entire sample period.

Next, we present the difference-in-differences empirical methodology used in the analysis. Then, we present the findings on the effect of QE1, QE2 and QE3 policies. To test the robustness of the findings, we conduct two additional tests in the Robustness Tests section. We then conclude the paper with a brief summary of the finding.

## 3 Empirical methodology

Exploiting the heterogeneity in the sensitivity to QE programs across banks, represented by cross-sectional variation in the MBS-to-Securities ratio, we use Difference-in-Differences strategy to identify the effect of QE programs. The dependent variables used are natural log of loans, loans as a share of total loans and log difference of loans, where loans studied are total loans, real estate loans, C&I loans and consumer loans. The baseline specification we use is given by,

(1) $$Y_{i,t}=\alpha +\beta_{1}MBS/Securities_{i}+\beta_{2}QE_{t}+\beta_{3}MBS/Securities_{i}\times QE_{t}+\beta_{4}X_{i,t}+e_{i,t}$$

where $Y_{i,t}=\{ln(y_{i,t}),\frac{y_{i,t}}{Total\;loans_{i,t}},dln(y_{i,t})\}$, where $y_{i,t}$ are the types of loans in consideration. $MBS/Securities_{i}$ is the 3-quarter average of MBS-to-Securities ratio of a bank prior to a QE. For robustness test, we divide the banks into treatment and control groups based on MBS-to-Securities ratio prior to a QE following Rodnyansky and Darmouni [35]. We replace $MBS/Securities_{i}$ with an indicator variable $treat_{i}$, where $treat_{i}$ is equal to 1 if a bank has MBS-to-Securities ratio greater than or equal to 75th percentile (treatment group) and 0 if MBS-to-Securities ratio less than or equal to 25th percentile (control group). Using the continuous variable, $MBS/Securities_{i}$, in the baseline model has the advantage of using all observations in the sample as opposed to using top 25 and bottom 25 percent of data only, based on MBS-to-Securities ratio, when using $treat_{i}$. Following the literature on bank lending (see Kashyap and Stein [26], Rodnyansky and Darmouni [35] for example), the matrix of control variables, $X_{i,t}$, includes size (natural log of assets), equity (normalized by total asset), reserves, currency, return on assets (ROA) and realized gains.^12^ Standard errors are clustered at the bank-level to allow for serial correlation across time.

The coefficient of interest is $\beta_{3}$ as it captures the difference in lending between banks with relatively high (treated) and low (untreated) MBS-to-Securities ratio after a QE, or the *treatment effect*. A positive $\beta_{3}$ hence implies that the treated banks issue more loans in question after a QE compared to their counterparts.

We use STATA software for the estimation. In particular, we use Difference-in-Differences package to estimate the parameters.^13^

**Figure 2:**
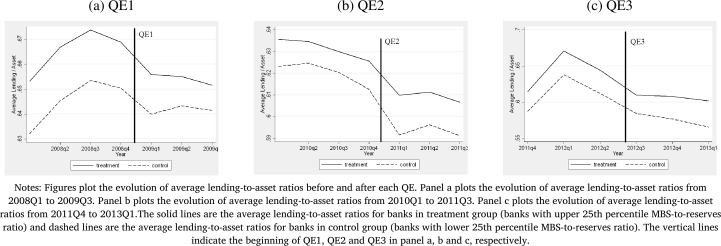
QE and Bank Lending.

One of the main challenges in assessing the impact of QE programs, particularly QE1, is the presence of confounding factors related to the Global Financial Crisis. As banks that specialize in real estate mortgages may have been disproportionately affected by the sharp decline in MBS prices, it can be difficult to separate any positive effects of QE programs from the negative effects of the crisis. This poses a challenge for disentangling the specific impacts of the QE programs on bank lending and risk-taking behavior from the broader economic conditions during the crisis. In particular, there are three potential limitations of the approach. First, the estimation approach assumes a parallel trend between the treatment and control groups in the absence of the treatment. The limitation of the difference-in-differences estimation approach is that we are unable to observe the trend of the treated group in the absence of treatment. Second potential concern that is closely related to the first concern in this case is the endogeneity of exposure. The MBS-to-securities ratio may be endogenous to bank lending and risk-taking behavior. Banks that are more willing to take risks may have intentionally held more MBS, anticipating the policy effects. Alternatively, banks with higher MBS holdings may have been more likely to receive LSAPs due to their size or relationship with the Federal Reserve. Given that QE programs were implemented to provide a general support to the housing market and improve financial condition, and considering the legal restriction governing assets purchases by the Fed, the cross-sectional variation in the MBS-to-Securities ratio is likely to be uncorrelated with the pre-intervention lending rates for each bank. Specialization in real-estate lending by some banks implies that the distribution of MBS-to-Securities ratio is unlikely to be random. However, while the levels of lending are different across banks with high and low MBS-to-Securities ratio throughout the sample period, the trend in lending is unlikely to be altered absent the Fed policies. In other words, the parallel trends assumption in lending across banks with different MBS-to-Securities ratios before the Fed policies is likely to hold. To further provide support to the parallel trend assumption and alleviate the concern of endogeneity, we analyze the trends in average lending-to-asset ratio across banks in the treatment and control groups. Figs. 2(a-c) plot the average lending-to-asset ratio across banks in treatment group (solid line) and across banks in control group (dashed line) before and after each round of QE. Panel a plots the average lending-to-asset ratio from 2008Q1 to 2009Q3 with the vertical line indicating the implementation of QE1, Panel b plots the average lending-to-asset ratio from 2010Q1 to 2011Q3 with the vertical line indicating the implementation of QE2 and Panel c plots the average lending-to-asset ratio from 2011Q4 to 2013Q1 with the vertical line indicating the implementation of QE3. While lending-to-asset ratio is different between two groups of banks across the time period in each graph, the trend differs only after the Fed's QE policies, providing support to the parallel trend assumption. In other words, before the onset of each QE, the trends in average lending-to-asset ratio are similar between banks in treatment and control group. To further analyze whether the banks in the treatment and control groups are fundamentally different, we calculate summary statistics across banks in each group for the year 2012Q2 in Table 2. As the table shows, the banks in the treatment group are similar to those in the control group in terms of deposits, liabilities, equities, currency and reserve holdings, realized gains, ROA and total lending. The banks in the treatment group are relatively larger in size and allot relatively larger share of total loans to real estate. Hence, they hold significantly more MBS compared to banks in control group as illustrated by the MBS-to-asset and MBS-to-Securities ratios in the table. Hence, the bank in these two groups appear to be different only in terms of composition of loans and MBS holdings but otherwise are fundamentally similar, providing support to the identification strategy.^14^ Third potential concern is the selection bias that the banks in the treatment group are systematically different than the banks in the control group. However, as shown above, the banks in the two groups are different only in terms of the MBS-to-asset and MBS-to-Securities ratios but otherwise similar.

**Table 2:** Summary Statistics of Banks in Treatment and Control Groups.

	Treatment Group		Control Group	
Variable	Mean	Standard Deviation	Mean	Standard Deviation
Size	12.72	1.54	11.42	1.08
Deposit / Asset	0.83	0.08	0.82	0.16
Liabilities / Asset	0.89	0.04	0.86	0.14
Equity / Asset	0.00	0.00	0.00	0.00
MBS / Asset	0.13	0.10	0.00	0.00
MBS / Securities	0.57	0.19	0.00	0.00
Treasury / Asset	0.00	0.01	0.02	0.07
Treasury / Securities	0.01	0.05	0.08	0.24
Currency / Asset	0.02	0.02	0.03	0.04
Reserves / Asset	0.08	0.07	0.11	0.11
Realized Gains / Asset	0.00	0.00	0.00	0.00
ROA	0.00	0.00	0.00	0.01
Total Loans / Asset	0.63	0.19	0.61	0.23
Real Estate Loans / Asset	0.47	0.18	0.41	0.21
C and I Loans / Asset	0.09	0.08	0.08	0.07
Credit Card Loans / Asset	0.00	0.06	0.00	0.02
Consumer Loans / Asset	0.00	0.01	0.00	0.00
Small Loans / Asset	0.17	0.12	0.21	0.15

Notes: The table reports the summary statistics of the variables in 2012Q2. Columns 2 and 3 show means and standard deviations of the variables for the commercials banks in the treatment group and columns 4 and 5 show means and standard deviations of the variables for the commercials banks in the control group.

In addition, the literature studying the QE policies document a narrow channel of the policies that affect the prices of the targeted asset solely and not through a broad channel that affect all long-term rates when targeted at a particular asset such as MBS, further providing support to the identification strategy.^15^ We also consider various robustness tests, including bank fixed effects to control for potential time-invariant fundamental differences across banks, to alleviate the concern of endogeneity.

Another limitation of the study is the generalizability of the findings to the overall financial system. The study focuses on commercial banks and may not be generalizable to other types of financial institutions. Nonetheless, understanding the effect on banks' behavior is an important finding.

There is a concern of borrower-level shocks still that could potentially affect the bank-level outcomes. For instance, banks with relatively high MBS holdings may experience higher credit demand growth due to improvement in their borrowers' financial health around the policy periods. To alleviate the concern, we refer the reader to Rodnyansky and Darmouni [35] and Kandrac and Schlusche [24], who match bank-level data with Dealscan loan-level data on C&I loans to estimate the effect of QE including borrower characteristics and find no significant effect of borrower-level shock on the bank-level lending outcomes.

## 4 Findings

**Figure 3:**
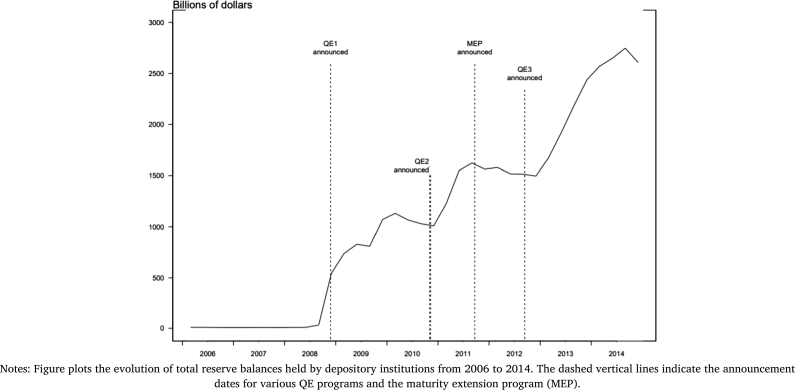
Total Reserves.

Until the Great Financial Crisis, the Fed relied on conventionary monetary policies that resulted in relatively modest increases in excess reserves in the banks' balance sheets. The QE policies implemented in response to the crisis and subsequent recession resulted in unprecedented increases in reserve balances held by banks. Fig. 3 plots the evolution of total reserves held by depository institutions from 2006 to 2014. Significant increases in the total reserves are observed after each round of QE. QE1 and QE3 specifically targeted MBS where the Fed purchased the banks' MBS holdings in exchange for reserves. Commercial banks held significant amount of MBS and as a result of the Fed policies, the banks experienced a significant increase in reserves held.^16^ Such increase in reserves likely affects the portfolio decisions, specifically the lending behavior, of the banks. We explore the effects and present the results below.

### 4.1 QE1

We present the findings in chronological order, starting with the first round of LSAP. QE1 began on November 25, 2008 when Federal Open Market Committee (FOMC) announced a program to purchase MBS with a stated goal of providing support to the housing market and improve financial condition more generally. By the end of 2010Q2, the program purchased a total of $1.25 trillion in agency MBS, $175 billion in federal agency debt issued by Fannie Mae, Freddie Mac and Ginnie Mae, and $300 billion in long-term treasury securities.

Table 3 reports the findings from estimating Equation (1) for the loans three quarters around 2008Q2. To save space, we present the estimated coefficient of interest ($\overset{ˆ}{\beta_{3}}$) only. Columns 1 through 4 present estimates using natural log of loans as the dependent variables, columns 5 through 8 use the loans as a share of total loans and columns 9 to 12 use growth rates of the loans. Columns 1, 5 and 9 do not include any controls, while columns 2, 6 and 10 include them. Columns 3, 7 and 11 include interactions of controls with QE1 indicator in addition to the controls. Columns 4, 8 and 12 include bank fixed effects to control for time-invariant characteristics in addition to the control variables. Row 1 presents analysis using MBS-to-Securities measure while row 2 uses the treatment indicator. Panels A to D present analysis on Total loans, Real Estate loans, C&I loans and Consumer loans respectively.

**Table 3:** Effect of QE1 on Lending.

	ln(y)				y / Total Loans				d ln(y)			
**Panel A: Total Loans**												
MBS-to-Securities x QE1	0.0412**	0.0617***	0.0641***	0.0670***	-	-	-	-	0.0311***	0.0312***	0.0294***	0.0403***
	(0.0177)	(0.0153)	(0.0166)	(0.0190)	-	-	-	-	(0.00880)	(0.00881)	(0.00971)	(0.0101)
treat x QE1	0.0422***	0.0558***	0.0534***	0.0561***	-	-	-	-	0.0224***	0.0224***	0.0232***	0.0286***
	(0.0144)	(0.0137)	(0.0162)	(0.0172)	-	-	-	-	(0.00688)	(0.00692)	(0.00812)	(0.00836)
Number of Observations	13952 / 6915	13952 / 6915	13952 / 6915	13952 / 6915	-	-	-	-	13929 / 6902	13929 / 6902	13929 / 6902	13929 / 6902

**Panel B: Real Estate Loans**												
MBS-to-Securities x QE1	0.0352**	0.0584***	0.0715***	0.0693***	0.00298	0.00412**	0.00564***	0.00368**	0.0587***	0.0595***	0.0840***	0.0662***
	(0.0161)	(0.0124)	(0.0139)	(0.0160)	(0.00192)	(0.00194)	(0.00216)	(0.00154)	(0.0123)	(0.0122)	(0.0137)	(0.0157)
treat x QE1	0.0328***	0.0470***	0.0547***	0.0541***	0.00315*	0.00397**	0.00503**	0.00385*	0.0405***	0.0410***	0.0682***	0.0458***
	(0.0118)	(0.00987)	(0.0114)	(0.0129)	(0.00161)	(0.00164)	(0.00206)	(0.00211)	(0.00941)	(0.00934)	(0.0118)	(0.0123)
Number of Observations	13869 / 6859	13869 / 6859	13869 / 6859	13869 / 6859	13952 / 6915	13952 / 6915	13952 / 6915	13952 / 6915	13845 / 6845	13845 / 6845	13845 / 6845	13845 / 6845

**Panel C: C and I Loans**												
MBS-to-Securities x QE1	0.0251	0.0440*	0.0492**	0.0472*	0.00148	0.00152**	0.00203**	0.00248**	0.0324***	0.0329***	0.0486***	0.0429***
	(0.0260)	(0.0236)	(0.0245)	(0.0252)	(0.00174)	(0.00074)	(0.00101)	(0.00121)	(0.0101)	(0.01000)	(0.0112)	(0.0121)
treat x QE1	0.0283	0.0394**	0.0448**	0.0293**	0.00212	0.00210**	0.000428	0.00282*	0.0280***	0.0284***	0.0122	0.0359***
	(0.0202)	(0.0189)	(0.0210)	(0.0141)	(0.00143)	(0.00103)	(0.00160)	(0.00137)	(0.00864)	(0.00856)	(0.0108)	(0.0101)
Number of Observations	13729 / 6774	13729 / 6774	13729 / 6774	13729 / 6774	13952 / 6915	13952 / 6915	13952 / 6915	13952 / 6915	13697 / 6752	13697 / 6752	13697 / 6752	13697 / 6752

**Panel D: Consumer Loans**												
MBS-to-Securities x QE1	0.0762***	0.0850***	0.0465***	0.0724***	0.0237***	0.0230***	0.00669*	0.0231***	0.0313***	0.0316***	0.0277***	0.0306***
	(0.0103)	(0.00928)	(0.0103)	(0.0156)	(0.00331)	(0.00333)	(0.00382)	(0.00492)	(0.00972)	(0.00970)	(0.0107)	(0.0104)
treat x QE1	0.0688***	0.064***	0.0302***	0.0686***	0.0199***	0.0195***	0.00673**	0.0196***	0.0200**	0.0203**	0.0120	0.0225**
	(0.0198)	(0.0183)	(0.0112)	(0.0209)	(0.00279)	(0.00280)	(0.00316)	(0.00417)	(0.00816)	(0.00814)	(0.00960)	(0.0106)
Number of Observations	13784 / 6809	13784 / 6809	13784 / 6809	13784 / 6809	13952 / 6915	13952 / 6915	13952 / 6915	13952 / 6915	13758 / 6794	13758 / 6794	13758 / 6794	13758 / 6794
Controls	No	Yes	Yes	Yes	No	Yes	Yes	Yes	No	Yes	Yes	Yes
Controls x QE1	No	No	Yes	No	No	No	Yes	No	No	No	Yes	No
Fixed Effect	No	No	No	Yes	No	No	No	Yes	No	No	No	Yes

Notes: The table presents coefficient estimates from specifications at the bank level relating average lending over three quarters before vs. after QE1 to a bank's initial exposure towards LSAPs, as captured by its MBS-to-Securities ratio or treatment indicator prior to QE1. The coefficients presented are the coefficients of the interaction term between MBS-to-Securities and QE1 in row 1 and between treatment indicator and QE1 in row 2. Columns 1 through 4 use natural log of loans as the dependent variable, columns 5 through 8 use ratio of the loans to total loans whereas columns 9 through 12 use log differential of the loans. Panels A through D present findings for Total loans, Real Estate loans, C and I loans and Consumer loans respectively. The controls include Size (log total assets), equity normalized by total assets, currency, reserves, return on assets (ROA) and realized gains on securities before QE1. Inclusion of controls, interaction of controls with QE1 and bank fixed effects are as indicated. Number of observations for analysis using MBS-to-Securities x QE1 are listed first (before “/”), followed by those for analysis using treat x QE1. All specifications include a constant and standard errors (in parentheses) are clustered at the bank-level to allow for serial correlation across time. ***,**,* indicate significance at the 1%, 5% and 10% levels, respectively.

The estimated coefficient of interest ($\overset{ˆ}{\beta_{3}}$), which measures the effect of the Fed's quantitative easing (QE) policy on bank lending, is positive and statistically significant across all specifications for total loans, suggesting that the banks' total lending increased in response to QE1. With the natural log of lending as the dependent variable, the estimates suggest that QE1 raised total lending of the treated banks by about 4 to 6% more relative to the banks with relatively low MBS-to-Securities ratio. With the growth rate of lending as the dependent variable, the estimates suggest that the growth in total lending by the treated banks increased by about 3 to 4% more relative to their counterpart.^17^ The estimates are statistically significant across all specifications, suggesting the robustness of the result. When using treatment indicator instead of MBS-to-Securities ratio in row 2, the estimates are slightly smaller but are statistically significant. Overall, the findings suggest that the total lending by banks with higher exposure towards the Fed's policy increased in response to QE1. When the general level of lending was down, the Fed's policy seems to have desired effect in terms of increased lending.

For riskier loans, the estimated coefficient of interest, $\overset{ˆ}{\beta_{3}}$, is positive and statistically significant for almost all specifications for Real Estate, C&I and Consumer loans, suggesting that the banks' lending in riskier category increased in response to QE1. With the natural logs of loans as the dependent variable, the estimates are higher for each category compared to estimates for total loans. Similarly, with the share of total loans as dependent variable, the estimates for the riskier loans are positive and significant. These two observations suggest that the banks issued more riskier loans in response to the increase in reserves due to QE1. The banks assumed more ex-ante risk in their lending portfolio by shifting towards the riskier loans. With the growth rates of lending as dependent variable, the estimates suggest that the growth in riskier loans is higher than that of total loans, suggesting a rapid increasing in the riskier loans compared to the total loans. Rapid increase in riskier loans may suggest ‘search for yield’ among banks who experience a reduction in net interest margin due to the influx of reserves as a result of the QE policy. When using treatment indicator instead of MBS-to-Securities ratio in row 2, the estimates are slightly smaller but are statistically significant in most cases.

The findings suggest that QE1 boosted not only the total lending by banks with higher exposure towards the Fed policy but also the share of loans in the riskier categories. The banks who benefited from the QE1 policy expanded on the riskier loans relative to their counterparts. Overall, the findings support the portfolio substitution effect and the risk-taking channel of the transmission of QE policy. While it may seem as though risk taking is a drawback of the Fed policy, Bernanke [6] notes that one of the objectives of both conventional and non-conventional monetary policies are to improve return to productive risk-taking. In that sense, the QE policy appears to have achieved the intended outcome.

### 4.2 QE2

Next, we present the findings on QE2. The program was initiated on November 3, 2010 and resulted in total purchases of $778 billion in long-term treasury securities by the end of 2011Q2. The program was implemented with a goal of alleviating deflationary concerns and stimulating the economy. The differentiating characteristics of QE2 is the purchase of treasury securities instead of MBS. Table 8 shows that the commercial banks' holdings of treasury securities are significantly less than that of MBS. Hence, if the effect of QE1 found above was due to a general effect, we expect the findings here to be similar to those of QE1. In fact, considering that the purchases during QE2 were of higher frequency and magnitudes, $75 billion each month, we expect to see a higher impact of QE2 on banks' lending behavior.

Table 4 reports the findings from estimating Equation (1) for the loans three quarters around 2010Q4. We present the estimated coefficient of interest ($\overset{ˆ}{\beta_{3}}$) only. Columns 1 through 4 present estimates using natural log of loans as the dependent variables, columns 5 through 8 use the loans as a share of total loans and columns 9 to 12 use growth rates of the loans. Columns 1, 5 and 9 do not include any controls, while columns 2, 6 and 10 include them. Columns 3, 7 and 11 include interactions of controls with QE2 indicator in addition to the controls. Columns 4, 8 and 12 include bank fixed effects to control for time-invariant characteristics in addition to the control variables. Row 1 presents analysis using MBS-to-Securities measure while row 2 uses the treatment indicator. Panels A to D present analysis on Total loans, Real Estate loans, C&I loans and Consumer loans respectively.

**Table 4:** Effect of QE2 on Lending.

	ln(y)				y / Total Loans				d ln(y)			
**Panel A: Total Loans**												
MBS-to-Securities x QE2	0.0322**	0.0144	0.0279	0.00388	-	-	-	-	0.00207	0.000638	0.00141	0.000296
	(0.0160)	(0.00959)	(0.0216)	(0.0104)	-	-	-	-	(0.00260)	(0.00250)	(0.00272)	(0.00356)
treat x QE2	0.0306**	0.00306	0.0183	0.00783	-	-	-	-	0.00267	0.00375	0.000779	0.00416
	(0.0151)	(0.0108)	(0.0129)	(0.0124)	-	-	-	-	(0.00427)	(0.00426)	(0.00304)	(0.00608)
Number of Observations	13694 / 7035	13684 / 7025	13684 / 7025	13684 / 7025	-	-	-	-	13685 / 7026	13680 / 7021	13680 / 7021	13680 / 7021

**Panel B: Real Estate Loans**												
MBS-to-Securities x QE2	0.0568***	0.00532	0.00734	0.0132	0.00886***	0.00410	0.0124	0.00317	0.000937	0.000888	0.00134	0.00134
	(0.0176)	(0.0101)	(0.0153)	(0.0103)	(0.00237)	(0.00280)	(0.00944)	(0.00207)	(0.00292)	(0.00281)	(0.00357)	(0.00406)
treat x QE2	0.0397***	0.00787	0.0111	0.0103	0.00787***	0.00242	0.0023	0.00367	0.00167	0.000450	0.000594	0.0000847
	(0.0151)	(0.00868)	(0.0120)	(0.00938)	(0.00214)	(0.00230)	(0.00413)	(0.00178)	(0.00239)	(0.00231)	(0.00242)	(0.00338)
Number of Observations	13616 / 6982	13606 / 6972	13606 / 6972	13606 / 6972	13694 / 7035	13684 / 7025	13684 / 7025	13684 / 7025	13610 / 6966	13605 / 6961	13605 / 6961	13605 / 6961

**Panel C: C and I Loans**												
MBS-to-Securities x QE2	0.00220	0.0422**	0.0237	0.000238	0.00603	0.00570	0.00558	0.00320	0.00718	0.00527	0.00310	0.00336
	(0.0239)	(0.0190)	(0.0204)	(0.0205)	(0.00710)	(0.00710)	(0.00713)	(0.00197)	(0.00646)	(0.00644)	(0.00688)	(0.00930)
treat x QE2	0.0167	0.0467***	0.0549***	0.0116	0.00510***	0.00507***	0.00473	0.00293	0.00827	0.00712	0.00706	0.00535
	(0.0205)	(0.0163)	(0.0178)	(0.0182)	(0.00154)	(0.00153)	(0.00356)	(0.00179)	(0.00546)	(0.00543)	(0.00595)	(0.00785)
Number of Observations	13494 / 6876	13485 / 6868	13485 / 6868	13485 / 6868	13694 / 7035	13684 / 7025	13684 / 7025	13684 / 7025	13488 / 6871	13483 / 6866	13483 / 6866	13483 / 6866

**Panel D: Consumer Loans**												
MBS-to-Securities x QE2	0.0762	0.0850	0.0465	0.0724	0.000604	0.000356	0.000818	0.000437	0.0221	0.0216	0.0116	0.0215
	(0.103)	(0.0928)	(0.103)	(0.156)	(0.000707)	(0.000713)	(0.000770)	(0.000960)	(0.0447)	(0.0447)	(0.0465)	(0.0755)
treat x QE2	0.0548	0.0594	0.0347	0.0442	0.000247	0.0000786	0.000467	0.0000491	0.0136	0.0132	0.00662	0.0130
	(0.0890)	(0.0815)	(0.0912)	(0.143)	(0.000627)	(0.000632)	(0.000682)	(0.000856)	(0.0394)	(0.0395)	(0.00418)	(0.0684)
Number of Observations	13784 / 6809	13784 / 6809	13784 / 6809	13784 / 6809	13952 / 6915	13952 / 6915	13952 / 6915	13952 / 6915	13758 / 6794	13758 / 6794	13758 / 6794	13758 / 6794
Controls	No	Yes	Yes	Yes	No	Yes	Yes	Yes	No	Yes	Yes	Yes
Controls x QE2	No	No	Yes	No	No	No	Yes	No	No	No	Yes	No
Fixed Effect	No	No	No	Yes	No	No	No	Yes	No	No	No	Yes

Notes: The table presents coefficient estimates from specifications at the bank level relating average lending over three quarters before vs. after QE2 to a bank's initial exposure towards LSAPs, as captured by its MBS-to-Securities ratio or treatment indicator prior to QE2. The coefficients presented are the coefficients of the interaction term between MBS-to-Securities and QE2 in row 1 and between treatment indicator and QE2 in row 2. Columns 1 through 4 use natural log of loans as the dependent variable, columns 5 through 8 use ratio of the loans to total loans whereas columns 9 through 12 use log differential of the loans. Panels A through D present findings for Total loans, Real Estate loans, C and I loans and Consumer loans respectively. The controls include Size (log total assets), equity normalized by total assets, currency, reserves, return on assets (ROA) and realized gains on securities before QE2. Inclusion of controls, interaction of controls with QE2 and bank fixed effects are as indicated. Number of observations for analysis using MBS-to-Securities x QE2 are listed first (before “/”), followed by those for analysis using treat x QE2. All specifications include a constant and standard errors (in parentheses) are clustered at the bank-level to allow for serial correlation across time. ***,**,* indicate significance at the 1%, 5% and 10% levels, respectively.

In contrast to the analysis of QE1, the estimated coefficients of interest, $\overset{ˆ}{\beta_{3}}$, are not statistically significant for almost any of the specifications, suggesting that QE2 did not significantly affect banks' lending behavior. The findings support the notion that QE works through a narrow channel that affects the prices of the targeted asset. Since the banks held significantly large amount of MBS in their books, the QE1 resulted in significant increase in reserves and hence, significant effect on their lending behavior. On the other hand, since the banks held relatively small amount of treasury securities, the effect of QE2 on banks' lending behavior was insignificant.

### 4.3 QE3

Lastly, we present the findings on QE3. The largely unanticipated round of LSAP began on September 13, 2012 that involved purchase of $40 billion in agency MBS per month initially. After the end of Operation Twist, where FOMC swapped its holding of short-term treasury securities with equal amount of long-term treasury securities in order to exert downward pressure on long-term rates, FOMC purchased $45 billion in long-term treasury securities per month in addition to the MBS. Starting December 2013, FOMC reduced its purchase to $75 billion per month. The program ended on October 29, 2014.

Table 5 reports the findings from estimating Equation (1) for the loans three quarters around 2012Q3. We present the estimated coefficient of interest ($\overset{ˆ}{\beta_{3}}$) only. Columns 1 through 4 present estimates using natural log of loans as the dependent variables, columns 5 through 8 use the loans as a share of total loans and columns 9 to 12 use growth rates of the loans. Columns 1, 5 and 9 do not include any controls, while columns 2, 6 and 10 include them. Columns 3, 7 and 11 include interactions of controls with QE3 indicator in addition to the controls. Columns 4, 8 and 12 include bank fixed effects to control for time-invariant characteristics in addition to the control variables. Row 1 presents analysis using MBS-to-Securities measure while row 2 uses the treatment indicator. Panels A to D present analysis on Total loans, Real Estate loans, C&I loans and Consumer loans respectively.

**Table 5:** Effect of QE3 on Lending.

	ln(y)				y / Total Loans				d ln(y)			
**Panel A: Total Loans**												
MBS-to-Securities x QE3	-0.00378	0.0208*	0.0242*	0.0255**	-	-	-	-	-0.00267	0.0257**	0.0342*	0.0263**
	(0.00454)	(0.0163)	(0.0174)	(0.0126)	-	-	-	-	(0.00202)	(0.0102)	(0.0176)	(0.0125)
treat x QE3	0.00397	0.0240**	0.0283*	0.0266**	-	-	-	-	0.00437	0.0437**	0.0296**	0.0274*
	(0.0140)	(0.0121)	(0.0151)	(0.0132)	-	-	-	-	(0.00421)	(0.0214)	(0.0142)	(0.0157)
Number of Observations	14061 / 7217	14061 / 7214	14061 / 7214	14061 / 7214	-	-	-	-	14008 / 7172	14008 / 7172	14008 / 7172	14008 / 7172

**Panel B: Real Estate Loans**												
MBS-to-Securities x QE3	-0.00623	0.0729***	0.0525**	0.0557**	0.00439**	0.00466**	0.00452**	0.00258**	0.0550***	0.0549***	0.0687***	0.0558**
	(0.00907)	(0.0271)	(0.0207)	(0.0282)	(0.00204)	(0.00187)	(0.00187)	(0.00127)	(0.0190)	(0.0191)	(0.0189)	(0.0268)
treat x QE3	-0.00636	0.0577*	0.0569**	0.0251*	-0.00299*	0.00273*	0.00293*	0.00205*	0.00236	0.0467**	0.0421*	0.0290**
	(0.0116)	(0.0294)	(0.0231)	(0.0131)	(0.00177)	(0.00158)	(0.00164)	(0.00122)	(0.00447)	(0.0223)	(0.0246)	(0.0139)
Number of Observations	13992 / 7159	13992 / 7156	13992 / 7156	13992 / 7156	14061/7217	14061/7214	14061/7214	14061/7214	13992 / 7156	13992 / 7156	13992 / 7156	13992 / 7156

**Panel C: C and I Loans**												
MBS-to-Securities x QE3	0.0469**	0.0333**	0.0411**	0.0356*	0.00385**	0.00438**	0.00537**	0.00250*	-0.0229	-0.0231	0.0310*	0.0274*
	(0.0225)	(0.0146)	(0.0208)	(0.0193)	(0.00173)	(0.00193)	(0.00269)	(0.00146)	(0.0174)	(0.0174)	(0.0184)	(0.0157)
treat x QE3	0.0202	0.0392**	0.0378**	0.0335**	0.00786***	0.00692***	0.00257**	0.00240**	-0.0118	0.0318**	-0.0163	0.0236**
	(0.0202)	(0.0168)	(0.0192)	(0.0139)	(0.00108)	(0.00109)	(0.00122)	(0.00101)	(0.0104)	(0.0134)	(0.0117)	(0.0114)
Number of Observations	13616 / 6906	13616 / 6903	13616 / 6903	13616 / 6903	14061/7217	14061/7214	14061/7214	14061/7214	13539 / 6840	13539 / 6840	13539 / 6840	13539 / 6840

**Panel D: Consumer Loans**												
MBS-to-Securities x QE3	-0.00566	0.0327**	0.0332**	0.0307**	0.0000465	0.00526*	0.00439*	0.00562**	0.0518**	0.0515**	0.0377**	0.0605*
	(0.0126)	(0.0158)	(0.0162)	(0.0156)	(0.0000312)	(0.00296)	(0.00258)	(0.00284)	(0.0244)	(0.0243)	(0.0177)	(0.0340)
treat x QE3	0.0243*	0.0713*	0.0340*	0.0272**	0.00626*	0.00583**	0.00345**	0.00520*	0.0325	0.0331**	0.0323	0.0362**
	(0.0146)	(0.0437)	(0.0193)	(0.0121)	(0.00331)	(0.00228)	(0.00154)	(0.00303)	(0.0247)	(0.0147)	(0.0282)	(0.0148)
Number of Observations	13616 / 6906	13616 / 6903	13616 / 6903	13616 / 6903	14061/7217	14061/7214	14061/7214	14061/7214	13539 / 6840	13539 / 6840	13539 / 6840	13539 / 6840
Controls	No	Yes	Yes	Yes	No	Yes	Yes	Yes	No	Yes	Yes	Yes
Controls x QE3	No	No	Yes	No	No	No	Yes	No	No	No	Yes	No
Fixed Effect	No	No	No	Yes	No	No	No	Yes	No	No	No	Yes

Notes: The table presents coefficient estimates from specifications at the bank level relating average lending over three quarters before vs. after QE3 to a bank's initial exposure towards LSAPs, as captured by its MBS-to-Securities ratio or treatment indicator prior to QE3. The coefficients presented are the coefficients of the interaction term between MBS-to-Securities and QE3 in row 1 and between treatment indicator and QE3 in row 2. Columns 1 through 4 use natural log of loans as the dependent variable, columns 5 through 8 use ratio of the loans to total loans whereas columns 9 through 12 use log differential of the loans. Panels A through D present findings for Total loans, Real Estate loans, C and I loans and Consumer loans respectively. The controls include Size (log total assets), equity normalized by total assets, currency, reserves, return on assets (ROA) and realized gains on securities before QE3. Inclusion of controls, interaction of controls with QE3 and bank fixed effects are as indicated. Number of observations for analysis using MBS-to-Securities x QE3 are listed first (before “/”), followed by those for analysis using treat x QE3. All specifications include a constant and standard errors (in parentheses) are clustered at the bank-level to allow for serial correlation across time. ***,**,* indicate significance at the 1%, 5% and 10% levels, respectively.

Findings are consistent with the findings and arguments for QE1. As both QE1 and QE3 resulted in relatively large increase in reserves for treated banks, they not only expanded their lending but also substituted towards making riskier categories of loans, providing evidence of the risk-taking channel.

The findings on these sections support the findings of Rodnyansky and Darmouni [35] and Kandrac and Schlusche [24] on the effect on total loans. The findings also suggest that banks increased the share of riskier loans in response to the reserve accumulation due to QE1 and QE3. The findings suggest that banks not only increase lending due to the policy but also engage in risky lending. The findings are also in line with those that study risk taking due to lower interest rates (see Maddaloni and Peydró [31], Jiménez et al. [23], Ioannidou et al. [22], Di Maggio and Kacperczyk [15], Dell'Ariccia et al. [14] and Heider et al. [21]).

The finding that QE1 and QE3 had a positive and significant impact on bank lending and the share of riskier loans is an important result with potentially significant implications for monetary policy. Specifically, the study provides evidence of a risk-taking channel through which LSAPs can affect bank behavior and the broader economy. One potential explanation for the findings on QE1 is that QE1 helped to alleviate the liquidity pressures faced by banks during the crisis, which in turn allowed them to increase lending and take on more risk. This interpretation is consistent with the stated goals of LSAPs, which were to increase the supply of credit and lower borrowing costs in order to support economic growth.

Another important implication of these findings is that they highlight the potential risks of LSAPs in terms of promoting excessive risk-taking by banks. While some degree of risk-taking is necessary for banks to provide credit and support economic growth, excessive risk-taking can lead to financial instability and systemic risk. Therefore, policymakers may need to carefully consider the potential trade-offs between the benefits and risks of LSAPs when making decisions about monetary policy.

Overall, the finding that QE1 had a positive impact on bank lending and risk-taking behavior provides important insights into the mechanisms through which LSAPs can affect the economy. However, it also raises important questions about the potential risks and unintended consequences of these policies, and highlights the need for continued research and careful consideration by policymakers.

## 5 Additional robustness tests

In this section, we further test the robustness of the main results by performing two additional tests. First, we estimate the baseline specification with large banks (banks in top 50% by total assets) only. This is to test if the main results are driven by the size effect. Second, we further test the validity of the methodology by estimating the baseline specification for quarters prior to QE3 policy. If the main results are biased, we expect the effects on lending behavior from the set of test should also be significant. Below, we present the results of these tests.

### 5.1 Size differential

While the baseline regressions control for the size of the banks, there's a possibility that the control is inadequate, perhaps due to nonlinearities. In order to alleviate the concern that the main findings are due to size effect, we restrict the sample to include banks in top 50% by total assets. Such restriction significantly reduces the size differential across banks and any potential size effect in the findings is reduced.

Table 6 presents the findings. We present the estimates for the coefficient of $MBS/Securities_{i}\times QE1$ only. For the sake of brevity, we present analysis on QE1 only.^18^ Columns 1 and 2 present estimates using natural log of loans as the dependent variables, columns 3 and 4 use the loans as a share of total loans and columns 5 and 6 use growth rates of the loans. Columns 1, 3 and 5 do not include any controls, while columns 2, 4 and 6 include them. Rows 1 to 4 present analysis on Total loans, Real Estate loans, C&I loans and Consumer loans respectively.

**Table 6:** Effect of QE3 on Lending.

	ln(y)		y / Total Loans		d ln(y)	
Total Loans	0.0277***	0.0266***	-	-	0.0208**	0.0208**
	(0.0087)	(0.0078)	-	-	(0.0104)	(0.0104)
Real Estate Loans	0.0545**	0.0578**	0.00577**	0.00578**	0.0325***	0.0327***
	(0.0266)	(0.0271)	(0.00275)	(0.00273)	(0.0115)	(0.0115)
C and I Loans	0.0313**	0.0328**	0.00585**	0.00586**	0.0308**	0.0308**
	(0.0130)	(0.0121)	(0.00250)	(0.00250)	(0.0154)	(0.0154)
Consumer Loans	0.0357**	0.0396***	0.00737**	0.00732**	0.0314***	0.0314***
	(0.0154)	(0.0150)	(0.00368)	(0.00368)	(0.0118)	(0.0118)
Controls	No	Yes	No	Yes	No	Yes

Notes: The table presents coefficient estimates from specifications at the bank level relating average lending over three quarters before vs. after QE3 to a bank's initial exposure towards LSAPs, as captured by its MBS-to-Securities ratio or treatment indicator prior to QE3. The analysis includes banks that are in upper 50 percentile based on size. The coefficients presented are the coefficients of the interaction term between MBS-to-Securities and QE3. Columns 1 and 2 use natural log of loans as the dependent variable, columns 3 and 4 use ratio of the loans to total loans whereas columns 5 and 6 use log differential of the loans. Rows 1 through 4 present findings for Total loans, Real Estate loans, C and I loans and Consumer loans respectively. The controls include Size (log total assets), equity normalized by total assets, currency, reserves, return on assets (ROA) and realized gains on securities before QE3. Inclusion of controls are as indicated. All specifications include a constant and standard errors (in parentheses) are clustered at the bank-level to allow for serial correlation across time. ***,**,* indicate significance at the 1%, 5% and 10% levels, respectively.

The estimates are similar to those of the baseline case. The findings suggest that the results are no different whether smaller banks are included in the analysis or not, ruling out the size effect explanation of our baseline results. The findings also suggest that there's no significant difference between large and small banks in lending sensitivity to the QE policy.

### 5.2 Timing of the effects

The set of tests aim to provide further support to the empirical methodology used in the paper. The biggest concern when estimating the effect of QE policy is that of endogeneity. In particular, some pre-existing trends might be driving the results as treated banks could have expanded lending prior to the QE policy relative to untreated banks. To alleviate such endogeneity concerns, we perform tests that make repeated observations of the banks over time. For brevity, we present analysis on QE3 only as the concern is most relevant to this round of QE.^19^ In particular, we study the banks three quarters prior to and following QE3. Following Granger [20], we estimate the following fixed-effects model. The series of tests allow us to ensure that the causes happen before consequences and not the other way around.

(2) $$Y_{i,t}=\gamma_{i}+\beta_{1}MBS/Securities_{i}+\beta_{2}D_{t}+\beta_{3}MBS/Securities_{i}\times D_{t}+\beta_{4}X_{i,t}+e_{i,t}$$

∀i,∀t∈{2011Q4,2012Q1,2012Q2,2012Q4,2013Q1,2013Q2}

where $Y_{i,t}$ are lending outcomes as defined in the baseline case, $\gamma_{i}$ are bank fixed effects, $X_{i,t}$ is the matrix of control variables and $D_{t}$ are indicators for time period, omitting 2012Q3. $MBS/Securities_{i}\times D_{t}$ represents the interaction of time indicator and the banks' sensitivity to QE3. Similar to above, $\beta_{3}$ is the coefficient of interest as it captures the difference in lending outcome between banks with relatively high and low MBS holdings over time. Studying the effect three quarters prior and following the QE allows us to analyze the causal direction and whether the effects grow or fade over time.

Table 7 presents the findings. We present estimated coefficient of interest ($\beta_{3}$) in Equation (2) only for three quarters prior and following QE3. Columns 1 through 6 present analysis for the quarters 2011Q4 to 2013Q2, omitting 2012Q3. Row 1 uses natural log of loans as the dependent variable, row 2 uses ratio of the loans to total loans whereas row 3 uses log differential of the loans. Panels A through D present analysis on Total loans, Real Estate loans, C&I loans and Consumer loans respectively.

**Table 7:** Timing of the Effects.

	2011Q4	2012Q1	2012Q2	2012Q4	2013Q1	2013Q2
**Panel A: Total Loans**						
ln(y)	0.00686	0.0263	-0.0266	0.0124**	0.0157***	0.0224*
	(0.00453)	(0.0202)	(0.0163)	(0.00523)	(0.00559)	(0.0120)
y / Total Loans	.	.	.	.	.	.
	.	.	.	.	.	.
d ln(y)	0.00950	0.0200	-0.0601	0.0117*	0.0111**	0.0335***
	(0.00644)	(0.0188)	(0.0541)	(0.00675)	(0.00560)	(0.0125)

**Panel B: Real Estate Loans**						
ln(y)	0.00784	0.0210	-0.0156	0.0473*	0.0321**	0.0412**
	(0.00609)	(0.0251)	(0.0201)	(0.0268)	(0.0144)	(0.0194)
y / Total Loans	-0.00174	0.00241	-0.00181	0.00631*	0.00720**	0.00801**
	(0.00158)	(0.00198)	(0.00143)	(0.00348)	(0.00332)	(0.00403)
d ln(y)	0.00892	0.0137	-0.0447	0.0325*	0.0408*	0.0432**
	(0.00738)	(0.0230)	(0.0430)	(0.0182)	(0.0214)	(0.0215)

**Panel C: C and I Loans**						
ln(y)	0.0186	0.0304	-0.0290	0.0302**	0.0505**	0.0415***
	(0.0123)	(0.0423)	(0.0336)	(0.0132)	(0.0256)	(0.0146)
y / Total Loans	0.00204	0.00361	0.00332	0.00742**	0.00798**	0.00821**
	(0.00131)	(0.00401)	(0.00389)	(0.00365)	(0.00372)	(0.00398)
d ln(y)	0.0218	0.00813	-0.0632	0.0543***	0.0307**	0.0358*
	(0.0161)	(0.0435)	(0.0701)	(0.0211)	(0.0134)	(0.0211)

**Panel D: Consumer Loans**						
ln(y)	-0.00331	0.0807	-0.0433	0.0205**	0.0675*	0.0441**
	(0.0459)	(0.0972)	(0.0836)	(0.0098)	(0.0391)	(0.0197)
y / Total Loans	-0.000175	0.0105	-0.00342	0.00518**	0.00654*	0.00584**
	(0.000232)	(0.0201)	(0.00831)	(0.00235)	(0.00387)	(0.00237)
d ln(y)	-0.00805	0.109	-0.118	0.0315**	0.0439*	0.118*
	(0.0756)	(0.106)	(0.160)	(0.0139)	(0.0267)	(0.0666)

Notes: The table presents coefficient estimates from specifications at the bank level relating lending three quarters before and after QE1 to a bank's initial exposure towards LSAPs, as captured by its MBS-to-Securities ratio prior to the time periods. The coefficients presented are the coefficients of the interaction term between MBS-to-Securities and the time periods. Row 1 uses natural log of loans as the dependent variable, row 2 uses ratio of the loans to total loans whereas row 3 uses log differential of the loans. Columns 1 to 6 present analysis for the time periods. Panels A through D presents findings for Total loans, Real Estate loans, C and I loans and Consumer loans respectively. The controls include Size (log total assets), equity normalized by total assets, currency, reserves, return on assets (ROA) and realized gains on securities before the time periods. All specifications include bank fixed effects and standard errors (in parentheses) are clustered at the bank-level to allow for serial correlation across time. ***,**,* indicate significance at the 1%, 5% and 10% levels, respectively.

The estimated coefficients are not significant for any quarters prior to QE3 while they are positive and significant after the implementation of QE3. The findings suggest no significance difference in lending behavior between treated and untreated banks before QE3 was phased in, and the treated banks expanded the loans relative to untreated banks after the adoption of the Fed policy, implying the causal effect of QE3 on banks' lending behavior. The findings provide further support to the identification strategy used in the paper.

In addition, there are evidences to show that the effects grow over time, indicating an increasing rate of shift towards riskier loans.

## 6 Conclusion

The paper explores the effect of bank-level reserve accumulation as a result of QE policies on bank lending and risk-taking behavior. Reserve creation by major banks has increased significantly on the onset of the Fed's response to the Great Financial Crisis, raising the question on role of such reserve accumulation on transmission of the QE policies. Despite rich theoretical literature on the role of reserve creation due to QE policy on bank lending behavior, empirical studies on such role is scarce. This paper contributes to the literature by studying the effect on banks' risk-taking behavior.

Exploiting the cross-sectional variation in the MBS holding in banks' portfolio, we employ a difference-in-differences identification strategy to estimate the effect of the QE policies on bank lending and risk-taking behavior. Each bank's exposure to the QE policies is captured by the MBS as a share of total securities in the bank's portfolio prior to the implementation of the policies. There's a significant heterogeneity in the MBS-to-securities ratio among the commercial banks and hence, their exposure to the QE policies.

We find that reserves created as a result of QE1 and QE3 led to banks with a relatively large fraction of MBS on their balance sheet to expand lending more aggressively when the Fed targeted those particular types of securities. Based on our findings, QE2 had no significant impact on banks' lending. The findings suggest that the transmission of QE works through a narrow channel that affect the prices of the targeted asset solely and not through a broad channel that affect all long-term rates. In addition to increased lending, the QE policies also led to an increase in the share of riskier loans in the banks' portfolios, supporting the existence of risk-taking channel.

There exist strong evidences that the overall efficacy of QE on banks' lending behavior depends on the types of assets purchased. The increase in reserves leads to an expansion of bank lending and shift towards riskier loans.

## CRediT authorship contribution statement

Saroj Dhital; Chandler Dixon; Eddie Evanczyk: Conceived and designed the experiments; Performed the experiments; Analyzed and interpreted the data; Contributed reagents, materials, analysis tools or data; Wrote the paper.

## Declaration of Competing Interest

The authors declare that they have no known competing financial interests or personal relationships that could have appeared to influence the work reported in this paper.

## Figures and Tables

**Table 8:** Summary Statistics.

Variable	Mean	Standard Deviation	Observations
Size	12.07	1.37	199,569
Equity / Asset	0.00	0.00	199,569
MBS / Asset	0.04	0.07	199,328
MBS / Securities	0.19	0.38	194,346
Treasury / Asset	0.01	0.04	199,328
Treasury / Securities	0.03	0.13	194,346
Currency / Asset	0.03	0.04	199,569
Reserves / Asset	0.09	0.10	199,569
Realized Gains / Asset	0.00	0.00	199,159
ROA	0.00	0.11	199,159
Total Loans / Asset	0.62	0.18	198,741
Real Estate Loans / Asset	0.45	0.19	198,741
C and I Loans / Asset	0.08	0.07	198,741
Credit Card Loans / Asset	0.00	0.04	198,741
Consumer Loans / Asset	0.02	0.04	198,741
Small Loans / Asset	0.14	0.13	198,741

Notes: The table reports the summary statistics of the variables. Columns 2, 3 and 4 show means, standard deviations and number of observations, respectively, of the variables for the commercials banks in the sample between 2008Q1 and 2014Q4.

## Data Availability

Data will be made available on request.

## Footnotes

1 The methodology to determine risk-weighted assets places assets into four risk-weighting categories: 0%, 20%, 50% and 100%. This weighting does not account for collateral, cash flow or character of the borrower.

2 Adrian and Shin [1] and Borio and Zhu [11] for instance, argue that banks take on larger risk as a result of lower interest rates. Here we focus on risk taking due to reserve expansion induced by QE.

3 See Friedman and Schwartz [17], Tobin [36], Bernanke and Reinhart [8], Andres et al. [3] for instance. Tobin [36], Bernanke and Reinhart [8] and Andres et al. [3] describe a mechanism where supply of an asset results in higher prices and lower yields on alternative assets, which induces public to hold the asset with new supply. Note, however, that the prediction relies on imperfect substitutability of the assets which, as pointed out by Krugman [30], can break when nominal interest rates are near or at zero.

4 With notable exception of Rodnyansky and Darmouni [35].

5 For example, Maddaloni and Peydró [31], Jiménez et al. [23], Ioannidou et al. [22], Di Maggio and Kacperczyk [15], Dell'Ariccia et al. [14] and Heider et al. [21].

6 See Bernanke and Gertler [7] and Kashyap and Stein [26] for instance.

7 See Puri et al. [34], Paravisini [32], Peek and Rosengren [33], Cornett et al. [12], Jiménez et al. [23] and Dagher and Kazimov [13].

8 See Bilgin et al. [10] for instance. Using a sample of 568 banks from 20 countries between 2009 and 2018, Bilgin et al. [10] find that economic uncertainty increases the default risk of conventional banks but does not affect Islamic banks' default risk. Our findings lend some support to their finding as the monetary policy response to an economic uncertainty in our analysis induces higher risk.

9 Also, following Kashyap et al. [27], we do not control for banks who engage in mergers and acquisition.

10 We take average of 3 quarters before QE3 (2011Q4 to 2012Q2).

11 Rodnyansky and Darmouni [35] finds a probability of switching from highest quartile to lowest quartile of MBS-to-Securities distribution to be 0.55% each quarter.

12 For robustness check, we also interact the controls with $QE_{t}$ to allow for potential heterogeneous responses to the QE by the banks.

13 Codes used for the analysis are available upon request.

14 Similar here implies that the differences are statistically insignificant.

15 See Krishnamurthy and Vissing-Jorgensen [29] for instance.

16 For instance, commercial banks held about 20 - 30% of total MBS outstanding during the period of 2013Q3 to 2014Q4 based on Federal Reserves' Flow of Funds.

17 The findings are similar qualitatively to those of Rodnyansky and Darmouni [35] but higher in magnitude.

18 We also drop the results from including the interactions of controls with QE1 and the bank fixed effects as they are similar to those presented in the table.

19 Results on QE1 are similar and are available upon request.

## References

[1] Adrian Tobias, Shin Hyun Song (2010). Handbook of Monetary Economics, vol. 3.

[2] Adrian Tobias, Shin Hyun Song (2010). Liquidity and leverage. J. Financ. Intermed..

[3] Andres Javier, López-Salido J. David, Nelson Edward (2004). Tobin's imperfect asset substitution in optimizing general equilibrium. J. Money Credit Bank..

[4] Bauer Michael, Rudebusch Glenn D. (2014). The signaling channel for federal reserve bond purchases. Int. J. Cent. Bank..

[5] Bernanke Ben, Gertler Mark (1989). Agency costs, net worth, and business fluctuations. Am. Econ. Rev..

[6] Bernanke Ben S. (2012).

[7] Bernanke Ben S., Gertler Mark (1995). Inside the black box: the credit channel of monetary policy transmission. J. Econ. Perspect..

[8] Bernanke Ben S., Reinhart Vincent R. (2004). Conducting monetary policy at very low short-term interest rates. Am. Econ. Rev..

[9] Bernanke Ben S., Gertler Mark, Gilchrist Simon (1999). Handbook of Macroeconomics, vol. 1.

[10] Bilgin Mehmet Huseyin, Danisman Gamze Ozturk, Demir Ender, Tarazi Amine (2021). Economic uncertainty and bank stability: conventional vs. Islamic banking. J. Financ. Stab..

[11] Borio Claudio, Zhu Haibin (2012). Capital regulation, risk-taking and monetary policy: a missing link in the transmission mechanism?. J. Financ. Stab..

[12] Cornett Marcia Millon, McNutt Jamie John, Strahan Philip E., Tehranian Hassan (2011). Liquidity risk management and credit supply in the financial crisis. J. Financ. Econ..

[13] Dagher Jihad, Kazimov Kazim (2015). Banks liability structure and mortgage lending during the financial crisis. J. Financ. Econ..

[14] Dell'Ariccia Giovanni, Laeven Luc, Suarez Gustavo A. (2017). Bank leverage and monetary policy's risk-taking channel: evidence from the United States. J. Finance.

[15] Di Maggio Marco, Kacperczyk Marcin (2017). The unintended consequences of the zero lower bound policy. J. Financ. Econ..

[16] D'Amico Stefania, King Thomas B. (2013). Flow and stock effects of large-scale treasury purchases: evidence on the importance of local supply. J. Financ. Econ..

[17] Friedman Milton, Schwartz Anna J. (1965). The State of Monetary Economics.

[18] Gagnon Joseph, Raskin Matthew, Remache Julie, Sack Brian (2018). The financial market effects of the federal reserve's large-scale asset purchases. 24th Issue (Mar 2011) of the Int. J. Cent. Bank..

[19] Gilchrist Simon, Zakrajšek Egon (2013). The impact of the federal reserve's large-scale asset purchase programs on corporate credit risk. J. Money Credit Bank..

[20] Granger C.W.J. (1969). Investigating causal relation by econometric and cross-sectional method. Econometrica.

[21] Heider Florian, Saidi Farzad, Schepens Glenn (2019). Life below zero: bank lending under negative policy rates. Rev. Financ. Stud..

[22] Ioannidou Vasso, Ongena Steven, Peydró José-Luis (2015). Monetary policy, risk-taking, and pricing: evidence from a quasi-natural experiment. Rev. Finance.

[23] Jiménez Gabriel, Ongena Steven, Peydró José-Luis, Saurina Jesús (2014). Hazardous times for monetary policy: what do twenty-three million bank loans say about the effects of monetary policy on credit risk-taking?. Econometrica.

[24] Kandrac John, Schlusche Bernd (2021). Quantitative easing and bank risk taking: evidence from lending. J. Money Credit Bank..

[25] Kashyap Anil K., Stein Jeremy C. (1994). Monetary policy and bank lending in monetary policy. Natl. Bur. Econ. Res. Stud. Bus. Cycles.

[26] Kashyap Anil K., Stein Jeremy C. (2000). What do a million observations on banks say about the transmission of monetary policy?. Am. Econ. Rev..

[27] Kashyap Anil K., Rajan Raghuram, Stein Jeremy C. (2002). Banks as liquidity providers: an explanation for the coexistence of lending and deposit-taking. J. Finance.

[28] Kiyotaki Nobuhiro, Moore John (1997). Credit cycles. J. Polit. Econ..

[29] Krishnamurthy Arvind, Vissing-Jorgensen Annette (2011).

[30] Krugman Paul (1998). It's baaack: Japan's slump and the return of the liquidity trap. Brookings Pap. Econ. Act..

[31] Maddaloni Angela, Peydró José-Luis (2011). Bank risk-taking, securitization, supervision, and low interest rates: evidence from the euro-area and the US lending standards. Rev. Financ. Stud..

[32] Paravisini Daniel (2008). Local bank financial constraints and firm access to external finance. J. Finance.

[33] Peek Joe, Rosengren Eric S. (2000). Collateral damage: effects of the Japanese bank crisis on real activity in the United States. Am. Econ. Rev..

[34] Puri Manju, Rocholl Jörg, Steffen Sascha (2011). Global retail lending in the aftermath of the US financial crisis: distinguishing between supply and demand effects. J. Financ. Econ..

[35] Rodnyansky Alexander, Darmouni Olivier M. (2017). The effects of quantitative easing on bank lending behavior. Rev. Financ. Stud..

[36] Tobin James (1969). A general equilibrium approach to monetary theory. J. Money Credit Bank..

